# Molecular and cellular basis of mu-opioid receptor signaling: mechanisms underlying tolerance and dependence development

**DOI:** 10.3389/fnins.2025.1597922

**Published:** 2025-06-24

**Authors:** Michael Swingler, Martina Donadoni, Ellen M. Unterwald, Sanjay B. Maggirwar, Ilker K. Sariyer

**Affiliations:** ^1^Department of Microbiology, Immunology and Inflammation, Center for Neurovirology and Gene Editing, Temple University Lewis Katz School of Medicine, Philadelphia, PA, United States; ^2^Department of Neural Sciences, Center for Substance Abuse Research, Temple University Lewis Katz School of Medicine, Philadelphia, PA, United States; ^3^Department of Microbiology, Immunology, and Tropical Medicine, School of Medicine and Health Sciences, The George Washington University, Washington, DC, United States

**Keywords:** opioids, tolerance, dependence, opioid receptors, signaling, alternative splicing

## Abstract

Opioids, while highly effective for pain management, are among the most addictive substances, contributing significantly to the global opioid crisis. Opioid use disorder (OUD) affects millions, with synthetic opioids like fentanyl exacerbating the epidemic due to their potency and widespread illicit availability. Opioids exert their effects through opioid receptors (ORs), primarily the mu opioid receptor (MOR), which mediates both therapeutic analgesia and adverse effects such as euphoria, dependence, and tolerance. Chronic opioid use leads to cellular adaptations, including receptor phosphorylation, desensitization, and recruitment of β-arrestin, which uncouple MOR from downstream signaling pathways. These changes, along with compensatory upregulation of adenylyl cyclase (AC) and cAMP signaling, underlie the development of tolerance, dependence, and withdrawal, however the exact signaling pathways responsible remain unknown. Emerging research highlights the role of neuroinflammation, genetic polymorphisms, and alternative splicing of MOR isoforms in modulating opioid responses and vulnerability to OUD. Current treatments for OUD, such as methadone, buprenorphine, and naltrexone, are limited by compliance, access, and relapse rates. Novel therapeutic strategies, including biased MOR agonists, opioid vaccines, and splice variant-specific agonists, offer promise for safer pain management and reduced abuse liability. However, a deeper understanding of opioid receptor signaling, neuroimmune interactions, and genetic factors is essential to develop more effective interventions. This review explores the molecular mechanisms of opioid tolerance, dependence, and withdrawal, emphasizing the need for innovative approaches to address the opioid crisis and improve treatment outcomes.

## Introduction

Opioids have been used for thousands of years for food, rituals, and medicinal purposes (Kritikos and Papadaki, 1967; Salavert et al., 2020). Opioids are the most widely used and effective medication for the treatment of pain; however, they are also one of the most addictive. It is estimated that over 6.1 million people age 12 or older in the USA have an opioid use disorder (OUD), and in 2022, 75% of reported drug overdose deaths in the USA were the result of an opioid (Center for Drug Evaluation and Research, 2024; National Institute on Drug Abuse, 2024). An opioid use disorder (OUD) is characterized by persistent, chronic use of opioids that results in significant impairment and continued use despite harmful consequences (CDC, 2024; Dydyk et al., 2024). Recent years have seen a spike in the use of synthetic opioids such as fentanyl, which is 50 and 100 more potent than heroin and morphine, respectively, (Ramos-Matos et al., 2024). The widespread illicit availability of fentanyl is exacerbating the opioid use epidemic, as other substances are being cut with fentanyl to modulate their effects.

Opioids can be classified as endogenous, natural, synthetic, or semi-synthetic; all of which are ligands for opioid receptors (ORs) (Lappas and Lappas, 2022). Endogenous opioids are those produced by the body (e.g., *β*-endorphin, enkephalin, dynorphin), natural opioids are those found naturally in the opium poppy *Papaver somniferum* (e.g., morphine, codeine), semi-synthetic opioids are derived from natural opioids (e.g., heroin, oxycodone, hydrocodone, buprenorphine), and lastly synthetic opioids are chemically synthesized and structurally unrelated to the natural alkaloids (e.g., fentanyl, methadone) (Marraffa, 2014; Hong et al., 2022; Lappas and Lappas, 2022). While all of these are agonists at ORs, they vary in structure which can alter their affinity and potency for the three ORs.

ORs are inhibitory G-protein coupled receptors (GPCRs), for which four different types have been identified; Mu (*μ*, MOR), kappa (*κ*, KOR), delta (*δ*, DOR), and nociceptin opioid receptor (NOP) (Dhawan et al., 1996; Cox et al., 2015; Toll et al., 2016; Lappas and Lappas, 2022). These receptors are expressed widely throughout various pain modulatory tissues in the body, including the brain, spinal cord, and peripheral nervous system, and are also found in the digestive tract (Yam et al., 2018). Agonists of ORs cause numerous effects, both therapeutic and adverse, including analgesia, euphoria/dysphoria, CNS depression, respiratory depression, nausea, constipation, and drowsiness (National Institute on Drug Abuse, 2021; Paul et al., 2021). The most common clinically used opioids, as well as the most commonly misused opioids, are agonists of the mu opioid receptor (MOR), which is encoded by the *OPRM1* gene (Wang et al., 1994). While activation of MOR results in analgesia, it also indirectly activates the central dopamine reward pathways producing euphoria and reward (Li et al., 2016; Steidl et al., 2017).

In this review, we discuss the current state of our understanding of mu opioid receptor signaling and pharmacology as it relates to opioid tolerance, dependence, and withdrawal. Additionally, we discuss the implications of the neuroimmune effects of opioids on the development of opioid dependence, as well as discuss how certain genetic modifications can play a role in opioid tolerance, dependence, and development of OUD, and, finally, emerging therapies for better treatment of OUD.

## The endogenous opioid system

The endogenous opioid system is responsible for a multitude of effects within both the central and peripheral nervous systems. The main classes of endogenous opioids are endorphins, enkephalins, dynorphins, and nociceptin, which are the primary endogenous agonists for MOR, DOR, KOR, and NOP, respectively, (Bodnar, 2022). These are peptides produced primarily by the brain, mainly the pituitary gland and hypothalamus, and the adrenal gland in response to many different types of stimuli including food, sex, and social interactions to regulate mood states (Darcq and Kieffer, 2018; Herman et al., 2024). The genes that encode the opioid peptide precursors are proopiomelanocortin (POMC), preproenkephalin (PENK), preprodynorphin (PDYN), and prepronociceptin (PNOC), which code for *β*-endorphin, the enkephalin peptides, the dynorphin peptides, and nociceptin, respectively. Following release, these peptides bind to opioid receptors, resulting in a variety of downstream signaling cascades based on receptor and peptide type and cellular expression.

The different types of ORs produce different downstream effects following activation. While MOR is the primary target for opioid analgesics, the DOR and KOR receptors also are involved in the modulation of pain. MOR activation is known to produce euphoria, while DOR activation is primarily associated with emotional state including positive affect and reduced anxiety, and KOR activation is associated with negative affect and dysphoria (Darcq and Kieffer, 2018; Valentino and Volkow, 2018). The activity of all of these receptors together contributes to the complexity of opioid mediated signaling.

## Opioid signaling

ORs are expressed in the central nervous system (CNS), peripheral nervous system (PNS), and gastrointestinal tract, with MOR being the most commonly expressed OR (Herman et al., 2024). MOR is encoded by the *OPRM1* gene, which is found on human chromosome 6 (Wang et al., 1994).

**Figure 1:**
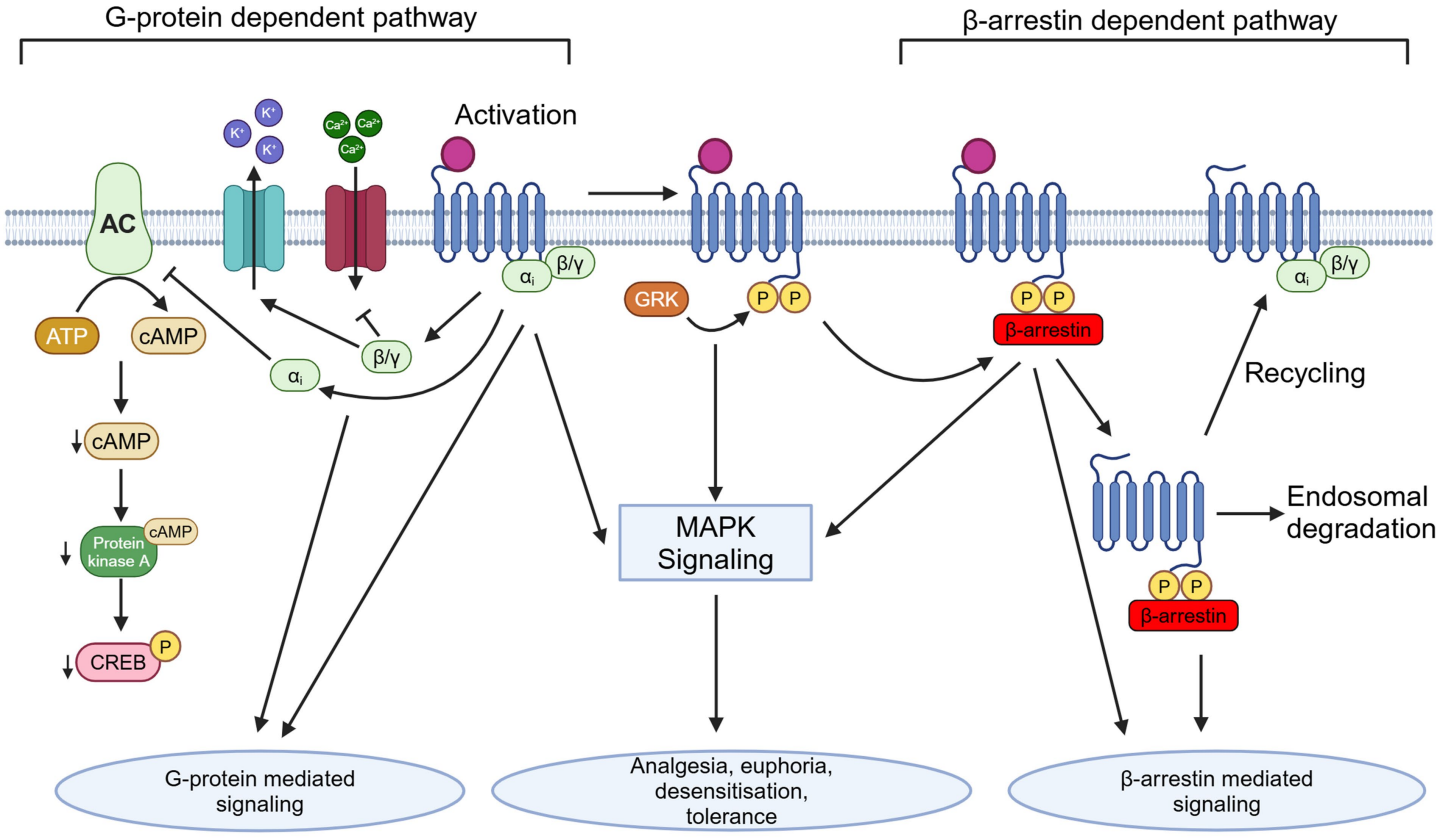
Depiction of G-protein dependent and β-arrestin dependent OR signaling pathways and downstream effects following activation, as well as depicting internalization and trafficking. AC, adenylate cyclase; ATP, adenosine triphosphate; cAMP, cyclic adenosine monophosphate; MAPK, mitogen-activated protein kinase; GRK, G-protein receptor kinase; P, phosphate group; Created in BioRender (https://BioRender.com/m15z911).

All types of opioid receptors are 7-transmembrane GPCRs with an extracellular binding domain and intracellular signaling domain (Clark et al., 2006; Manglik et al., 2012; Dhaliwal and Gupta, 2024). ORs are coupled to inhibitory G-proteins, Gαi and Gαo. Upon receptor activation, the Gαi/o and Gβγ subunits are released and act on a variety of downstream intracellular pathways (Hilger et al., 2018). This G-protein signaling is the “classical” opioid signaling pathway, however it is only one of the signal transduction pathways activated by opioids. In addition to activation of G-protein signaling, the β-arrestin signaling pathway is also activated (Figure 1; Al-Hasani and Bruchas, 2011; Kee et al., 2024). This β-arrestin signaling pathway is thought to be involved in the regulation of opioid signaling through receptor desensitization and internalization (Al-Hasani and Bruchas, 2011).

The Gai subunit released by OR agonist binding inhibits adenylyl cyclase (AC) on a cellular level. AC is responsible for converting ATP to cyclic adenosine monophosphate (cAMP). This results in a decrease in intracellular cAMP levels, thus a decrease in activation of cAMP-dependent protein kinase A (PKA), which is responsible for phosphorylation and activation of downstream proteins, such as cAMP response element binding protein (CREB). Phosphorylated CREB binds to cAMP response element (CRE) promoter, resulting in transcription of genes downstream of cAMP. The Gβγ subunits released act differently at a presynaptic and postsynaptic level on neurons activated by opioids. Presynaptically, the Gβγ subunit will bind to voltage-gated calcium channels (VGCCs) and inhibit them, preventing release of neurotransmitters. On the other hand, postsynaptic Gβγ subunits activate G protein-coupled inward-rectifying potassium (GIRK) channels, which prevents depolarization by releasing K^+^ from the cell (North et al., 1987).

Important downstream signaling includes MAPK signaling pathways such as ERK, JNK, and p38, however the mechanisms are ligand and receptor specific (Gutstein et al., 1997; Gendron et al., 2016; Gopalakrishnan et al., 2022). This downstream signaling occurs in response to phosphorylation of the C-terminal domain of the OR following agonist binding. The internal C-terminal domain of MOR contains several serine, threonine, and tyrosine phosphorylation sites, however the exact roles that these sites play in the function of MOR signaling remains unclear (El Kouhen et al., 2001; Schulz et al., 2004; Kelly, 2011). Additionally, the exact downstream effects of these activated kinases on development of opioid dependence and tolerance remains unknown. Furthermore, recent studies have shown that different ligands can induce different patterns of phosphorylation on the same type of GPCR (Butcher et al., 2011; Doll et al., 2011). For example, it has been shown that low efficacy agonists such as morphine produce selective phosphorylation of the MOR-Ser375 residue without phosphorylation of other residues within the motif, which results in lower β-arrestin recruitment. In contrast, high efficacy agonists such as fentanyl promote more robust phosphorylation of MOR-Ser375, as well as Thr370, Thr376, and Thr379, which promotes greater β-arrestin recruitment (Just et al., 2013; Underwood et al., 2024).

Following opioid receptor phosphorylation, β-arrestin is recruited to the C-terminal of the receptor. β-arrestin is a scaffolding protein whose main role is as a key regulator of GPCR activity. It has been shown that following opioid receptor binding to an agonist, various sites within the C-terminal domain of the receptor are phosphorylated by GPCR kinases (GRKs) (Retamal et al., 2019). β-arrestin binds to these phosphorylated points and facilitates desensitization and internalization. After the receptor has been internalized, it is either degraded by the lysosome or dephosphorylated in the endosomal compartment by phosphatase enzymes and returned to the cell surface in a process called resensitization (Kelly, 2005; Drake et al., 2006; Mohan et al., 2012; Badal et al., 2018).

Opioid receptor agonism can have differential effects depending on the specific type of cell it is acting on. While MOR activation in the CNS mainly results in modulation of neuronal activity, it also has effects on glial cells such as microglia and astrocytes (Hutchinson et al., 2007; Watkins et al., 2009). The main effects of MOR activation on neurons are the inhibition of neurotransmitter release, primarily inhibiting GABAergic neurons, and activation of downstream MAPK signaling. The direct inhibition of GABAergic neurons can result in further effects on other neurotransmitters. In contrast, on glial cells, opioids are known to have neuroimmune modulatory effects and result in activation of glial cells, causing release of cytokines such as TNF-*α*, IL-1β, and IL-6 (Cuitavi et al., 2023).

## Cellular mechanisms of opioid tolerance

Tolerance can be defined as the decreased response to a drug following prolonged or repeated exposure (Pietrzykowski and Treistman, 2008). This can be seen in the context of therapeutic or recreational drug usage, where an increase in dose is needed after a time to maintain its initial effect. Prolonged exposure to a drug can also lead to physiological dependence, which is not the same as tolerance. Physiological dependence can be described as physiological adaptations to prolonged or repeated exposure to a drug in which discontinuation will result in withdrawal symptoms (Griffin, 1990; Szalavitz et al., 2021). It is important to note that physiological dependence is not the same as addiction, nor is physical dependence required for addiction. Addiction is defined as a compulsive drive to continue taking a drug despite adverse consequences and is a complex behavior with a multitude of genetic, physical, psychological, and environmental factors playing a role (Volkow and Li, 2004). *In vitro*, drug tolerance and dependence can be studied, as there are molecular markers showing these effects. However, addiction cannot be measured *in vitro* or in cell culture, since it is a measurement of behavioral effects, not only physiological. If an individual is dependent on an opioid, they will undergo withdrawal following its discontinuation. Withdrawal is associated with changes at the molecular level. The physiological signs of opioid withdrawal include aches and pain, muscle spasms, cramps, nausea, vomiting, diarrhea, anxiety, insomnia, sweating, as well as other adverse effects (Vernon et al., 2016; Kosten and Baxter, 2019).

Perhaps, the most obvious idea in terms of the cause of developing opioid tolerance would be downregulation in the number of ORs, and this has been reported following chronic administration of some opioid agonists (Stafford et al., 2001). However, studies have revealed that downregulation of ORs is inconsistent between different opioid agonists and therefore, may not completely explain tolerance. For example, Stafford et al. examined the contribution of down regulation of MOR in mice treated with morphine or etorphine, another opioid agonist (Stafford et al., 2001). They observed a substantial decrease in MOR levels in mice treated with etorphine, but little to no changes in mice treated with morphine. Following these observations, it is currently believed that, instead of OR downregulation being the only mechanism of tolerance development, OR are also desensitized and become uncoupled from downstream signaling pathways (Waldhoer et al., 2004). Thus, there are receptor density-dependent and -independent mechanisms underlying the development of opioid tolerance. The exact molecular mechanisms underlying opioid tolerance and dependence *in vivo* are still unclear, however research points toward the regulation of opioid receptors via the mechanisms of desensitization, phosphorylation, β-arrestin recruitment, internalization, and recycling to be involved in the development of opioid tolerance and dependence (Figure 1; Dang and Christie, 2012; Raehal and Bohn, 2014; Bowman and Puthenveedu, 2015; Zhou et al., 2021). Desensitization of a GPCR is a complex process that results in reduced receptor signaling in response to an agonist after repeated stimulation and may be largely responsible for the development of opioid receptor tolerance and dependence (Connor et al., 2004, 2015; Rajagopal and Shenoy, 2017).

Acute opioid exposure results in a decrease in AC activity and a decrease in cAMP levels within cells (Sharma et al., 1975a,b). However, it is also revealed that following chronic opioid exposure, the cells exhibit a compensatory increase in AC activity (Sharma et al., 1975a,b, 1977). This increase in AC activity was first seen after morphine withdrawal was induced in morphine treated cells by adding naloxone, a MOR antagonist, or by washing the cells (Sharma et al., 1975a). Following withdrawal from opioids, there is a spike in cAMP levels due to the overactivity of AC. This is known by a variety of names such as cAMP overshoot, supersensitization, or superactivation (Sharma et al., 1975a,b, 1977). This compensatory increase in AC activity, which can be observed via cAMP overshoot and superactivation, is hypothesized to be one of the key aspects responsible for the development of opioid tolerance and dependence, as well as facilitating the negative effects associated with opioid withdrawal (Sharma et al., 1975a; Madhavan et al., 2010). Since the discovery of this superactivation of the cAMP pathway, it has been described as the most significant molecular adaptation in response to chronic opioids (Bie et al., 2005). It has been shown that this cAMP overshoot can result in an increase in GABAergic input to the dopamine neurons within the VTA (Meye et al., 2012). As a result, analyzing the cAMP pathway in response to opioids is one of the most important tools in measuring opioid tolerance, dependence, and withdrawal *in vitro* and *in vivo*.

The cAMP overshoot phenomenon was first shown by Sharma et al. (1975a,b). The cAMP overshoot in an opioid dependent *in vitro* system may be precipitated by the addition of an antagonist such as naloxone, or by removing the agonist through washing the cells. Xia et al. developed a high-throughput cell based assay model to measure morphine-induced cAMP overshoot (Xia et al., 2011). They used this screen to identify 24 inhibitors of cAMP overshoot in response to opioid withdrawal, showing how this assay may be useful in identifying compounds that can inhibit morphine-induced dependence, withdrawal, and addiction. The overshooting of the cAMP pathway in response to opioid withdrawal is a transient event, with cAMP levels being shown to peak 15–30 min after withdrawal *in vitro* (Xia et al., 2011). As such, it is important to be able to monitor cAMP levels at these time points. A common way that researchers have looked at levels of cAMP in an *in vitro* setting is through methods such as enzyme linked immunosorbent assays (ELISAs) to directly measure cAMP levels, as well as fluorescence based assays targeting various points within the cAMP pathway, radioimmunoassays, and other new methods to measure cAMP levels in live cells (Sprenger and Nikolaev, 2013). In addition to the use of direct measurements of cAMP, it is possible to target the downstream effectors of cAMP by measuring CREB or PKA phosphorylation. As phosphorylation of these effector proteins correlates to their activation in response to cAMP levels, it is possible to measure the phospho-levels of these proteins compared to their total levels and correlate that to changes in cAMP levels within the cells (Guitart et al., 1992; Lane-Ladd et al., 1997; Ren et al., 2013; Pena et al., 2018). Assessment of levels of these phosphorylated proteins is a common method for examining cAMP activity in tissues collected from rodent studies.

Since the discovery of cAMP overshoot, research has focused on how specific compounds or receptors may prevent opioid dependence by inhibiting this phenomenon. Wen et al. (2012) investigated the mechanism by which cholecystokinin octapeptide (CCK-8), a potent endogenous anti-opioid, exerts its effects (Faris et al., 1983; Wen et al., 2012). They used cAMP overshoot to demonstrate that the CCK1 receptor is responsible for the inhibitory effects of CCK-8 on morphine dependence (Wen et al., 2012). These observations were confirmed when Hao et al. (2018) demonstrated that the overexpression of CCK1 receptor prevented cAMP overshoot in HEK293-hMOR cells, treated with morphine, as well as preventing phosphorylation of CREB and ERK1/2, suggesting that CCK1R overexpression blocked morphine dependence in this system (Hao et al., 2018).

One of the next most important mechanisms thought to be involved in the development of opioid tolerance and dependence is the recruitment of β-arrestins to ORs in response to agonism (Finn and Whistler, 2001; Al-Hasani and Bruchas, 2011; Zhou et al., 2021). The recruitment of β-arrestins to phosphorylated C-terminal domains of ORs results in receptor desensitization and endocytosis, causing signaling termination and decreases in downstream responses (Ferguson, 2001; Luttrell and Lefkowitz, 2002; Surratt and Adams, 2005; Lau et al., 2011). Desensitization of MOR is a result of uncoupling of the receptor from its G protein, which is initiated by β-arrestins (Connor et al., 2015; Kee et al., 2024). However, the desensitization of MOR does not directly correlate with the subsequent internalization of receptor. While certain opioids such as endorphins and methadone result in receptor desensitization and endocytosis by β-arrestins, these processes are less robust in response to morphine (Finn and Whistler, 2001; Lau et al., 2011). This is believed to be an example of biased agonism of MORs (Finn and Whistler, 2001; Lau et al., 2011). Biased agonism is the difference in activation of various kinases and subsequent β-arrestin recruitment in response to different opioids binding MOR (Finn and Whistler, 2001; Lau et al., 2011). Research into biased MOR agonism has become a major point of focus in recent years, as the differences in activation pathways between different opioid agonists could play a major role in development of tolerance and dependence, as well as become a target of new treatments for OUD.

## Brain region specific responses

As stated above, most clinically useful opioid analgesics, as well as opioids used recreationally, are MOR agonists. MORs are expressed in circuits involved in pain transmission including primary afferent neurons in the periphery (i.e., Aδ and C fibers), spinal cord dorsal horn neurons and central thalamic neurons (Kimmey et al., 2022). MOR activation in these circuits is responsible for the pain-relieving actions of opioid analgesics. Opioids also activate MORs in limbic brain areas including the ventral tegmental area (VTA), nucleus accumbens, and striatum which participate in opioid reward, reinforcement, and tolerance (Williams et al., 2013; Adhikary and Williams, 2022). Opioid receptor signaling cascades vary across brain regions and cellular location. The culmination of opioid actions in the CNS are due to both their pre- and post-synaptic effects. Postsynaptic opioid actions include, as discussed above, inhibition of adenylyl cyclase and increased potassium channel opening with resultant reductions in cell excitability and neuronal hyperpolarization. At presynaptic sites, opioids close voltage-gated calcium channels and thus inhibit neurotransmitter release. Through these mechanisms, opioids reduce the transmission of painful signals from the periphery through the spinal cord and to the brain, producing their characteristic analgesic effects. As related to opioid reinforcement, opioid receptor-dependent inhibition of GABA release from interneurons in the VTA results in activation of the mesolimbic dopamine reward pathway through a disinhibition mechanism (Gendron et al., 2016; Charbogne et al., 2017; Cooper et al., 2017; Serafini et al., 2020). Through this indirect mechanism, opioids increase dopamine release in the nucleus accumbens, producing their other characteristic effect, euphoria.

Chronic exposure to opioids can result in opioid tolerance and dependence. Although these phenomena are well established, the mechanisms and brain regions involved are not clearly elucidated (Adhikary and Williams, 2022; Gamble et al., 2022). Opioid tolerance may be mediated by alterations in MOR signaling and trafficking. As described previously, chronic opioid exposure is associated with a downstream adaptive mechanism involving the compensatory upregulation of adenylyl cyclase activity and associated cAMP-dependent signaling (Sharma et al., 1975a,b). Upon opioid removal, overshoot in the production of cAMP occurs resulting in increased activation of PKA and associated downstream events. The cell populations involved in this response have been studied to a limited extent and mostly through studies performed *in vitro* or *ex vivo*. For example, cAMP overshoot and upregulation of cAMP-dependent signaling in response to opioid withdrawal has been shown in VTA slices of opioid-dependent animals, an effect that results in increased GABA transmission (Bonci and Williams, 1997; Madhavan et al., 2010). In addition, injection of Rp-cAMPS, an inhibitor of cAMP-dependent PKA activation, directly into the VTA of morphine-dependent rats attenuates naloxone precipitated withdrawal symptoms (Madhavan et al., 2010). These studies support the role of altered cAMP signaling within the VTA in opioid dependence. Other studies have focused on neurons in the locus coeruleus (LC) and their role in cellular opioid tolerance (Adhikary and Williams, 2022). The LC plays a central role in autonomic and stress responses, including those induced by opioid withdrawal. Noradrenergic neurons in the LC become hyperactive during opioid withdrawal, contributing to withdrawal symptoms such as restlessness, anxiety, sweating, and tachycardia. In brain slices containing LC neurons of animals treated chronically with morphine, acute application of morphine enhances MOR desensitization and signal uncoupling as compared with the effect in untreated animals (Dang and Christie, 2012; Adhikary and Williams, 2022). The degree of desensitization and tolerance varies depending on opioid agonist potency and efficacy and on the degree of phosphorylation of the MOR C-terminus (Arttamangkul et al., 2019; Adhikary and Williams, 2022). The phenomenon of MOR desensitization is thought to underlie the maintenance of cellular opioid tolerance.

## Neuroinflammation and its contribution to tolerance and dependence

While discussing neuroinflammatory responses to opioids, it is important to acknowledge its dual role in modulating the general immune response, including immunosuppressive, as well as immunostimulatory effects. Opioids, particularly morphine and heroin, have been shown to dampen various immune functions. This includes the downregulation of Natural Killer (NK) cell activity, T and B cell responses, antibody formation, and phagocytic activity of neutrophils and macrophages. These cells are responsible for phagocytosis, apoptosis regulation, cytokine and chemokine release, antibody formation, as well as a variety of other tightly controlled immunological processes (Bonilla and Oettgen, 2010; Turvey and Broide, 2010; Marshall et al., 2018). Downregulation of these cell types result in immunosuppression, which increases risk of infection and pathogenesis. Opioids have been shown to decrease the production of cytokines and chemokines by immune cells such as macrophages, microglia and astrocytes, further contributing to immunosuppression (Lefkowitz and Chiang, 1975; Bussiere et al., 1993; Núñez and Urzúa, 1999; Eisenstein, 2019). The immunosuppressive effects of opioids are primarily mediated through MOR activity in immune cells (Gavériaux-Ruff et al., 1998; Núñez and Urzúa, 1999; McCarthy et al., 2001; Boland and Pockley, 2018; Plein and Rittner, 2018; Eisenstein, 2019). Consistently, the epidemiological studies have linked high doses and the initiation of opioid therapy with a higher risk of infections, such as pneumonia, due to impaired immune function (Plein and Rittner, 2018). However, not all opioids share the same immunomodulatory properties. For instance, buprenorphine has been shown to have a more favorable immunological profile compared to morphine and fentanyl, particularly in cancer patients, buprenorphine and tramadol treatment may have less detrimental or even beneficial effects on immune function (Franchi et al., 2007; Boland and Pockley, 2018). Some studies also suggest that morphine can stimulate the immune system via binding to MD2, a molecule associated with Toll-like Receptor 4 (TLR4) (Alexander and Rietschel, 2001; Hutchinson et al., 2010; Zhang et al., 2020; Gabr et al., 2021; Thomas et al., 2022). TLR4 plays a crucial role in the innate immune response, where its activation drives the release of proinflammatory cytokines and transcriptional activation (Newton and Dixit, 2012; Gabr et al., 2021). However, this hypothesis of opioid immune activation through TLR-4 is debated, as morphine is generally found to be immunosuppressive even in TLR4-deficient models (Hutchinson et al., 2010; Eisenstein, 2019). Immune cells are also known to secrete endogenous opioid peptides that bind to peripheral opioid receptors, potentially relieving inflammatory and neuropathic pain (Celik et al., 2016). Together, this indicates a complex interaction where opioids can also have immunostimulatory effects (Celik et al., 2016; Plein and Rittner, 2018).

TLR4-mediated neuroinflammation in the periaqueductal gray (PAG) drives opioid tolerance through soluble TNF signaling. Blocking TNF signaling thus can prevent neuroinflammation and preserves morphine efficacy (Eidson et al., 2017). Morphine-induced hyperalgesia and tolerance are also associated with increased adenosine kinase expression and reduced A3 adenosine receptor (AR) signaling. Enhancing A3AR signaling with agonists was found to attenuate these adverse effects by reducing neuroinflammation (Doyle et al., 2020). Similarly, both morphine and fentanyl activate the NLRP3 inflammasome in glial and neuronal cells, leading to neuroinflammation and tolerance. Suppression of NLRP3 inhibits tolerance and prevent hyperalgesia (Carranza-Aguilar et al., 2022). Thus, the interplay between MOR, TLR4, A3AR, and their composite effect on neuroinflammation appears to be critical in the development of opioid tolerance and dependence.

Outside of the CNS, peripheral monocytes play a significant role in opioid tolerance and dependence, primarily through their impact on immune function and chemotaxis. Opioids such as heroin and morphine significantly block the chemotactic response of monocytes, a crucial function in immune response, presumably via activation of mu and delta opioid receptors in a manner that can be reversed by naloxone, contributing to the functional defects observed in intravenous drug users (Pérez-Castrillón et al., 1992). However, transmigration of monocytes across the BBB is elevated during opioid use, for example an activation of glial and immune cells leads to the production of proinflammatory mediators, creating neuroinflammatory state that is fundamental in the transition from acute to chronic pain. This neuroinflammation also disrupts the analgesic effects of opioids, contributing to the tolerance and dependence and the migration of peripheral monocytes into the central nervous system (CNS) (Echeverria-Villalobos et al., 2023). Opioids like morphine induce the release of extracellular vehicles from astrocytes, which contains miR-23a. This miRNA leads to the loss of pericyte coverage at the blood–brain barrier (BBB), increasing the influx of peripheral monocytes into the CNS and promoting neuroinflammation (Liao et al., 2022). Opioids, and other recreational drugs, increase the frequency of CD14^low^CD16^high^ (non-classical) monocytes in the peripheral blood and their translocation into the CNS. Elevated dopamine levels, common during substance use, further enhance this transmigration, exacerbating neuroinflammation and contributing to neurocognitive impairments (Calderon et al., 2017). Chronic morphine exposure in mice, especially in the context of HIV-1 infection, facilitates the trafficking of inflammatory monocytes (Ly6C+) and T-cells (CD3+) into the CNS, driven by chemokine gradients and TLR activation (Dutta and Roy, 2015). Chemokines like CCL2 (aka MCP-1) play a crucial role in the transmigration of non-classical monocytes across the BBB, which is dramatically reduced the CCL2-mediated monocyte transmigration, suggesting a potential dual benefit in reducing neuroinflammation and treating opioid addiction (Dutta and Roy, 2015; Jaureguiberry-Bravo et al., 2018).

## Molecular and genetic aspects influencing dependence, tolerance, and withdrawal

**Figure 2:**
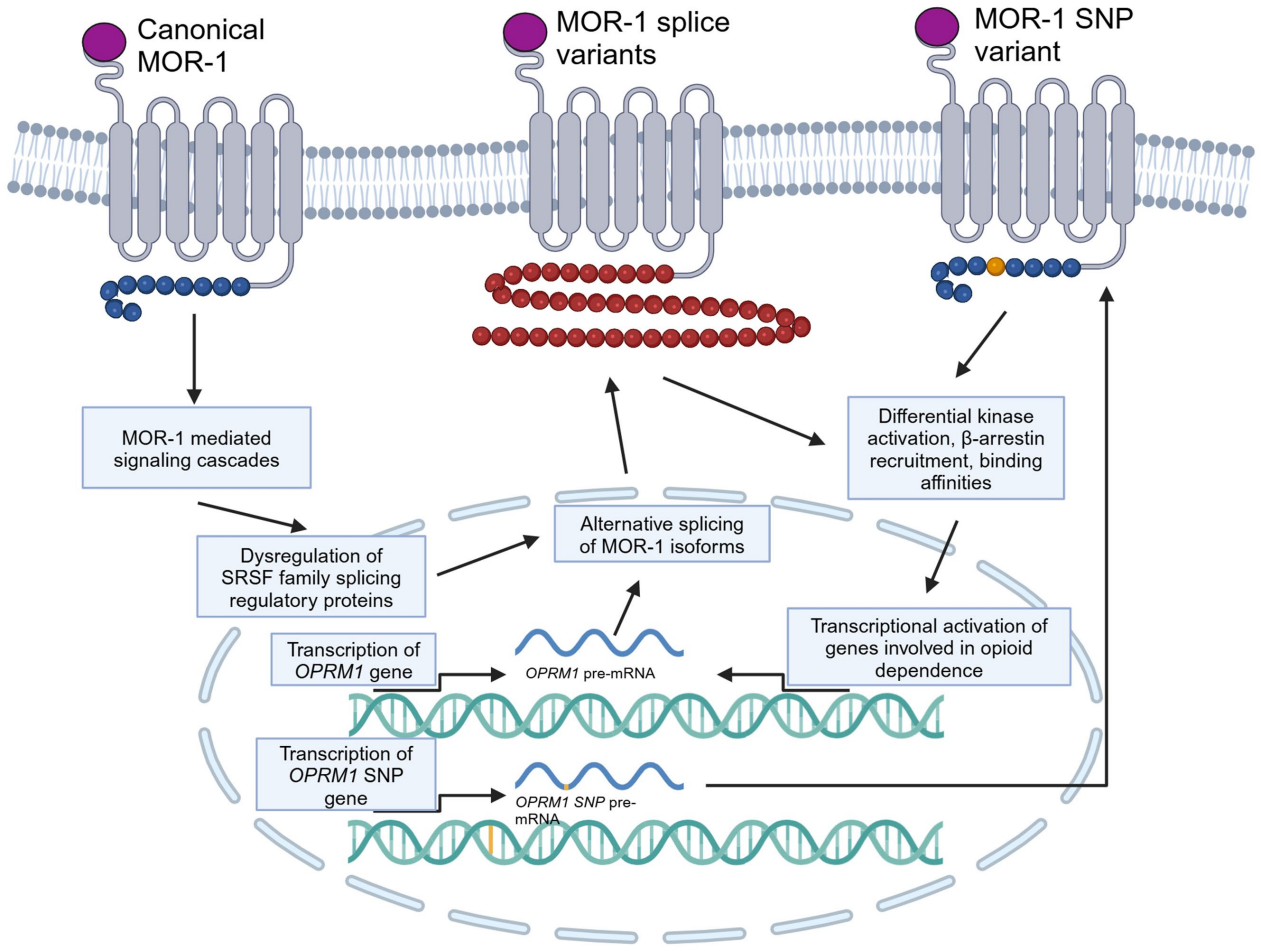
Molecular and genetic alterations of MOR, specifically alternative splicing and SNPs, and how they may be involved in dependence and tolerance signaling. This cartoon depicts a generically alternatively spliced 7TM C-terminal variant of MOR and a MOR receptor with an SNP on the C-terminal. Created in BioRender (https://BioRender.com/p97q223).

While downstream effects of opioid signaling can result in dependence, tolerance, and addiction, there are certain genetic responses that can occur that may be implicated in the development of tolerance and dependence (Figure 2). One such change that could be linked to the development of cellular tolerance is the alternative splicing of the *OPRM1* gene. There have been 20 alternatively spliced isoforms of human MOR identified, with the MOR-1 variant being the most studied and defined as the canonical MOR receptor due to it being the most expressed form of the receptor (Pan et al., 1999, 2003, 2005; Pasternak and Pan, 2013; Williams et al., 2013; Regan et al., 2016; Abrimian et al., 2021; Liu et al., 2021). These isoforms can be full length seven transmembrane (7TM) GPCRs, truncated 6TM which lack a N-terminal binding domain, or even further truncated 1TM receptors. All 7TM spliced variants have different C-terminal domains compared to MOR-1 (Abrimian et al., 2021). The different C-terminals domains of the 7TM isoforms are shown in Table 1. The C-terminus of MOR is phosphorylated following activation and is responsible for downstream signaling and β-arrestin recruitment. Since these spliced variants contain different C-terminals than MOR-1, they may have different phosphorylation sites, resulting in different downstream signaling cascades and different abilities to recruit β-arrestin. As a result, the isoforms may result in activation of downstream dependence-related genes in a different manner than canonical MOR-1. Differential downstream gene activation may also provide insight into what specific pathways play a role in the development of opioid tolerance and withdrawal.

**Table 1:** MOR 7TM isoform C-terminal tail protein sequences.

7TM isoforms	C-term	NCBI ref seq
MOR-1	REFCIPTSSNIEQQNSTRIRQNTRDHPSTANTVDRTNHQLENLEAETAPLP	NP_000905
MOR-1A	REFCIPTSSNIEQQNSTRIRQNTRDHPSTANTVDRTNHQVRSL	NP_001008504
MOR-1A2	REFCIPTSSNIEQQNSTRIRQNTRDHPSTANTVDRTNHQNYYIIHRCCCNTPLISQKPVLLWFCD	NP_001272452
MOR-1B1	REFCIPTSSNIEQQNSTRIRQNTRDHPSTANTVDRTNHQKIDLFQKSSLLNCEHTKG	NP_001138754
MOR-1B2	REFCIPTSSNIEQQNSTRIRQNTRDHPSTANTVDRTNHQRERRQKSDW	NP_001138755
MOR-1B3	REFCIPTSSNIEQQNSTRIRQNTRDHPSTANTVDRTNHQGPPAKFVADQLAGSS	NP_001138756
MOR-1B4	REFCIPTSSNIEQQNSTRIRQNTRDHPSTANTVDRTNHQS	NP_001138757
MOR-1B5	REFCIPTSSNIEQQNSTRIRQNTRDHPSTANTVDRTNHQVELNLDCHCENAKPWPLSYNAGQSPFPFPGRV	NP_001138758
MOR-1X	REFCIPTSSNIEQQNSTRIRQNTRDHPSTANTVDRTNHQCLPIPSLSCWALEQGCLVVYPGPLQGPLVRYDLPAILHSSCLRGNTAPSPSGGAFLLS	NP_001008505
MOR-1O	REFCIPTSSNIEQQNSTRIRQNTRDHPSTANTVDRTNHQPPLAVSMAQIFTRYPPPTHREKTCNDYMKR	NP_001008503
MOR-1i	REFCIPTSSNIEQQNSTRIRQNTRDHPSTANTVDRTNHQLENLEAETAPLP	NP_001138751

While these spliced variants are expressed at a low level compared to MOR-1 at standard physiological conditions, they have been shown to be upregulated in response to various stimuli, such as acute and chronic morphine treatment, as well as exposure to methadone and heroin (Vousooghi et al., 2009; Verzillo et al., 2014; Xu et al., 2015; Regan et al., 2016; Brown et al., 2022; Donadoni et al., 2022). Specifically, the MOR-1X isoform has been shown to be upregulated in cell lines following acute and chronic application of morphine, and is associated with differential activation of kinases such as ERK1/2 and p90 RSK1/2 when compared to the MOR-1 isoform (Regan et al., 2016; Donadoni et al., 2022). With this, it was also noted that morphine treatment increased splicing regulatory factor SRSF1, which may play a role in increasing the rate of MOR-1X alternative splicing (Regan et al., 2016). The MOR-1X isoform contains a unique C-terminal domain with additional phosphorylation sites for kinases such as protein PKA, which plays a key role in the cAMP pathway. Additionally, MOR-1X has been shown to be upregulated in the medial prefrontal cortex of male human heroin users, as well as in cortex tissues obtained from people with HIV (Brown et al., 2022; Donadoni et al., 2022). Chronic morphine exposure additionally upregulates MOR variants MOR-1B2 and MOR-1C1, which has been shown to shift G protein coupling from inhibitory to stimulatory signaling, contributing to tolerance (Chakrabarti et al., 2021). The different MOR isoforms also exhibit different binding affinities for different classes of opioids, including both endogenous and exogenous ligands (Pan et al., 2003, 2005). As a result, different opioids may result in different downstream signaling by preferentially inducing and binding to specific isoforms. Isoform specificity to different opioids and differences in downstream signaling may play a role in MOR biased agonism. This idea was recently explored by Narayan et al. (2021), who showed differences between G protein activation and β-arrestin recruitment between six different mouse MOR splice variants, each with unique C-terminal domains (Narayan et al., 2021).

In addition to the potential roles alternatively spliced 7TM MORs may have in opioid signaling, there has been some work looking at the effect of truncated 6TM receptors. In mouse studies, it has been revealed that the 6TM receptors are essential for opioid analgesia and may be targets for novel opioids that lack side effects (Majumdar et al., 2011; Lu et al., 2018). Using these mouse models, a novel compound, IBNtxA, has been identified as a potent 6TM MOR agonist that can produce analgesia. Mice that have knock out (KO) for all 6TM isoforms show morphine analgesia but lack IBNtxA analgesia. In contrast, when mice have KO for all 7TM isoforms, they lack morphine analgesia but retain IBNtxA analgesia. Mice with double KO for 6TM and 7TM receptors do not respond to either morphine or IBNtxA (Majumdar et al., 2011; Lu et al., 2018). While these studies were performed using mouse models, they provide novel insight into potential actions of 6TM MOR isoforms in humans.

Beyond splice variants, there also have been certain polymorphisms of ORs that have been linked to opioid tolerance and dependence. A specific single nucleotide polymorphism (SNP) within the *OPRM1* gene, named SNP rs1799971 (A118G), is a prominent target for study due to its association with OUD. Several studies have shown an association between *OPRM1* A118G and development of heroin addiction (Shi et al., 2002; Bart et al., 2004; AL-Eitan et al., 2021). It was also shown that the A118G variant binds *β*-endorphin with three times the affinity than the standard MOR variant, and that β-endorphin is three times more potent at GIRK activation in the A118G form than the unaltered form (Bond et al., 1998). Furthermore, the A118G polymorphism has shown an increase in responsiveness to naltrexone in humans with alcohol dependence, as well as an decrease in buprenorphine efficacy in a murine model of OPRM1 A118G (Chamorro et al., 2012; Browne et al., 2017). This suggests that differing polymorphisms in the *OPRM1* gene may be associated with different risks for the development of OUD, as well as being involved in treatment efficacy. While A118G polymorphism is the only SNP that has been extensively studied, there may be more of an implication for of polymorphisms on an individual’s vulnerability for the development of OUD. Another study suggests that genetic modulation, such as single point mutation at the MOR T394 phosphorylation site, blocks opioid tolerance and increased vulnerability to heroin self-administration further validating MOR polymorphisms on dependence, as well as identifying as a potential therapeutic target (Wang et al., 2016).

Outside of the context of OUD, an SNP has been identified in women of Chinese descent and is associated with fentanyl-induced emesis. Women undergoing gynecological surgery were genotyped for SNPs in the *OPRM1* gene, from which an SNP in the MOR-1X isoform was identified that is associated with an individual being 5.6 times more likely to develop fentanyl-induced emesis in a postoperative setting (Pang et al., 2012).

## Clinical treatment of OUD

There are three FDA-approved medications for the treatment of OUD, buprenorphine, methadone, and naltrexone. Methadone is an opioid receptor full agonist that can assist in mitigating opioid withdrawal and craving. Buprenorphine is an opioid receptor partial agonist that binds with high affinity to MOR receptors (Greenwald et al., 2014). It reduces craving and is an effective treatment option. Both drugs maintain tolerance to opioid-induced respiratory depression and hence protect patients if they return to opioid use. By binding to the MOR, they also diminish the effects of an illicit opioid that might be consumed. Naltrexone, in contrast, is a long-acting opioid receptor antagonist that can be used to treat OUD. Naltrexone will prevent an opioid from binding to and activating MOR if the person returns to drug use. In this way, the positive effects of opioid use are diminished, and the person is protected from a potential overdose. There are some limitations to these treatments, namely compliance, access, stigma, and abuse liability of methadone and buprenorphine. These medications are most effective when combined with psychosocial and/or behavioral therapies. Despite these treatment options, relapse rates remain high, ranging from 80 to 90% within the first year after treatment (Smyth et al., 2010).

In light of these observations, finding more efficacious treatments for OUD is a crucial area of focus. Current research has explored a variety of different routes to develop better interventions for OUD. One area of interest is the development of opioid vaccines (Bremer and Janda, 2017; Haile et al., 2022). These vaccines can bind to the opioids in the periphery and prevent them from being able to cross the BBB into the CNS, thus inhibiting their rewarding and reinforcing effects. Haile et al. (2022) developed a vaccine against fentanyl and showed that the vaccine blocked the effects of fentanyl, but not morphine, in both male and female rats. Additionally, they showed that the antibodies generated from their vaccine were specific to fentanyl and sufentanil, a fentanyl derivative, however were not specific to morphine, methadone, buprenorphine, or oxycodone (Haile et al., 2022). As this vaccine is specific to fentanyl, it would allow the vaccinated person to still be administered the other clinically used opioids mentioned, and as fentanyl has become the main illicit opioid being used, this would prevent the effects of majority of illicility obtained opioids in the event of a relapse (Haile et al., 2022; Ramos-Matos et al., 2024). However, this is a vaccine which may have implications in immunocompromised individuals, which many people with OUD are. The safety and efficacy of a vaccine like this in humans still needs to be explored, however could be a powerful preventative measure for people with OUD.

Another potential strategy is to create safer opioids to use in clinical settings to prevent the development of OUD. One such area of research is utilizing biased agonists which theoretically would produce analgesia with reduced reinforcing effects. An example of a biased agonist is oliceridine (TRV734), which was approved by the FDA in 2020. Oliceridine is a biased MOR agonist with selectivity for the G protein signaling pathway, with reduced β-arrestin signaling which typically is associated with opioid adverse effects. Oliceridine showed a decreased risk for adverse effects such as constipation and respiratory depression. While oliceridine is an effective analgesic, it has similar abuse liability to other opioid analgesics both in human and animal studies (Negus and Freeman, 2018). Newer G-protein biased MOR agonists are under development as better alternatives to oliceridine (Schmid et al., 2017). These compounds have higher degrees of bias than oliceridine, however their abuse potential needs further study.

Agonists with specificity to MOR splice variants have the potential to be effective analgesics that lack reinforcing properties. As mentioned previously, IBNtxA has been identified as a MOR 6TM specific agonist that produces analgesia in rodent models (Majumdar et al., 2011; Lu et al., 2018). Further study into splice variant-specific agonists could result in the development of safer opioid medications for pain management.

While our rodent models for studying the effects of opioids can provide novel insight into behavior, the cellular effects do not completely mimic that of a human brain due to differences in genomes. As a result, models that better represent the human brain can provide new understandings of human specific effects of opioids at the cellular level without the need for post-mortem brain tissue. The rise of induced pluripotent stem cell (hiPSC) derived 2D and 3D-cell culture models in recent years has given us a powerful tool to model the human brain *in vitro* through the use of primary derived neurons and cerebral organoids (hCOs). Researchers have now used hCOs to model the effects of opioids on the developing brain to elucidate the impacts of maternal opioid use (Yao et al., 2020; Dwivedi and Haddad, 2024). hCOs have also been used to model the role that MOR receptors have on pain signaling mechanisms (Fernandes et al., 2022). iPSCs derived from patients with OUD have been used to generate hCOs and neurons, and have been shown to respond to opioids (Sheng et al., 2016; Unterholzner et al., 2021; Ho et al., 2024). The use of these models can allow us to examine how genetic variations play a role in the development of opioid dependence.

## Conclusion

While opioids have been used for thousands of years, there is still much we do not know about the mechanisms underlying the development of opioid tolerance, dependence, and addiction (Pasternak and Pan, 2013). In order to further elucidate these mechanisms, there are many routes that may be explored. One area that should be further explored is the implications of alternatively spliced isoforms on downstream opioid mediated signaling. The differences between spliced isoform signaling in response to opioids may unveil novel targets for treatments of OUD or the development of safer opioid medications. Another area that should be further explored is the mechanisms of biased opioid signaling. The mechanisms underpinning biased opioid signaling may not only expand our understanding of opioid mediated signaling and effects of different exogenous opioids, but it may also provide useful insights into potential therapeutic targets. Expanding upon biased opioid signaling, the development of opioids for pain and analgesia that lack euphoric, reinforcing, and/or rewarding effects by biasing toward specific pathways or specific receptor types may allow the development of medications with less abuse liability than those currently offered.

To further elucidate opioid signaling mechanisms in the CNS, the use of human cerebral organoids as a model can provide novel insights (Notaras et al., 2021; Li et al., 2024). This is especially important due to the immunomodulatory effects of opioids on glial cells, which will release cytokines upon activation that induce changes in other cell types such as neurons. While there are rodent models to study these effects, they lack the human genome, so observations in rodents may not translate to humans. As such, there is a need to establish better models of human opioid effects in the lab setting.

In summary, there are many different directions to explore with opioid addiction, but it all comes down to this; we need a better understanding of opioid receptor signaling and how opioids produce tolerance and dependence. We also need to further understand how the proposed mechanisms involved in opioid tolerance and dependence interact with each other, since it is clear that no single system or pathway is solely responsible for the development of these opioid-related adaptations.
